# Four-step conversion of a declared seasonal coefficient of performance into hourly, capacity-aware coefficient-of-performance profiles for certified air-to-water heat pumps

**DOI:** 10.1016/j.mex.2026.104120

**Published:** 2026-08-28

**Authors:** Karl-Villem Võsa, Alo Mikola, Jarek Kurnitski, Raimo Simson

**Affiliations:** a Department of Civil Engineering and Architecture, Tallinn University of Technology (TalTech), Ehitajate tee 5, Tallinn, 19086, Estonia; b Department of Civil Engineering, Aalto University, P.O. Box 12100, Espoo, FI-00076, Finland

**Keywords:** Heat pump, Air-to-water heat pump, SCOP, COP curve, EN 14825, Heat Pump Keymark, Building energy simulation, Bivalent operation, Backup heating, Python implementation

## Abstract

Dynamic building-energy simulation requires a heat-pump coefficient of performance (COP) at hourly or sub-hourly time steps, while product certificates and energy-performance calculations often provide only a declared seasonal coefficient of performance (SCOP). This article describes a four-step method for converting a declared EN 14825 SCOP into an hourly, capacity-aware COP profile for certified air-to-water heat pumps with sufficient certificate data. First, a heating curve maps outdoor temperature to supply-water temperature and temperature lift; for the Colder rating zone this curve coincides with the outlet-water conditions of the rating itself. Second, a population COP(ΔT) model fitted to Heat Pump Keymark certificate data, validated out-of-sample on held-out manufacturers, provides the hourly COP shape within its fitted lift range of about 11–77 K. Third, an anchoring factor rescales this shape so that the modelled system reproduces the declared SCOP over the EN 14825 rating bins — an internal-consistency guarantee of the conversion, with the shape validity assessed separately. Fourth, capacities declared on the certificate split the load between heat-pump operation and electric backup. The method is independent of any particular simulation tool and serves as a pre-processor for dynamic building simulation, building-stock analysis, or energy-performance assessment. Fitted coefficients, a two-stage worked example with expected outputs, automated tests, and a standalone Python implementation are provided in a versioned Zenodo deposit.

• Converts a single declared SCOP into an hourly, capacity-aware COP profile for building simulation.
• Anchoring reproduces the declared SCOP as an internal-consistency guarantee; the COP shape is validated out-of-sample on held-out manufacturers.
• Requires only standard EN 14825 certificate quantities; a standalone Python implementation, fitted coefficients and automated tests are provided.

## Graphical abstract

**Figure:**
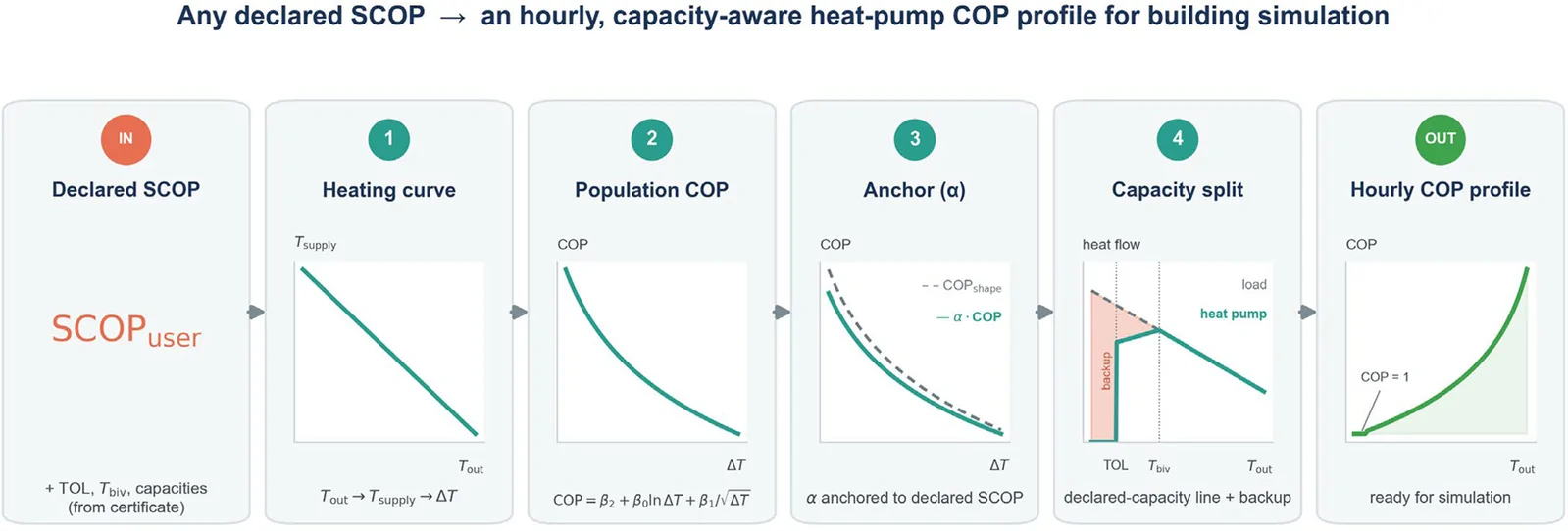

**Specifications table**

**Table:** 

**Subject area**	Energy
**More specific subject area**	Building energy performance simulation; heat-pump seasonal performance modelling
**Name of your method**	Four-step conversion of a declared SCOP into an hourly, capacity-aware COP profile using a population COP model
**Name and reference of original method**	The method combines standard components — EN 14825 [1] bin integration, a population COP(ΔT) shape fitted to certified Heat Pump Keymark test points, seasonal anchoring to the declared SCOP, and a capacity-based backup split from declared certificate quantities. The contribution is their integration on a large, deduplicated public certified population and the resulting reproducible, certification-anchored pre-processor for building-energy simulation.
**Resource availability**	Fitted model coefficients (all-data and cold-only variants), the EN 14825 bin templates, the two-stage worked example with expected outputs and automated tests, validation scripts, and a standalone Python implementation are provided in a versioned Zenodo deposit [2]: https://doi.org/10.5281/zenodo.22029915 (CC BY 4.0; cite the release version matching this article). Source certificates: Heat Pump Keymark database, https://keymark.eu/ [3].

## Background

Heat pumps are central to European building decarbonisation, and their real efficiency in cold climates is one of the key uncertainties of that transition [4,5]. Policy and practice represent this efficiency almost exclusively through seasonal metrics: ecodesign and energy-labelling regulations rate products by a seasonal coefficient of performance (SCOP) determined under EN 14825 [1], and the energy-performance-certificate (EPC) frameworks implementing the Energy Performance of Buildings Directive [6] fall back on tabulated default seasonal values when no product has yet been selected — the Estonian calculation methodology is a typical example [7]. Dynamic building-energy simulation, however, needs a coefficient of performance (COP) that varies hour by hour with the operating conditions — primarily the lift between the supply-water and outdoor temperatures — and time-resolved heat-pump operation is equally central to energy-system and demand-flexibility studies [8].

The gap between the declared seasonal scalar and the hourly characteristic is normally bridged ad hoc. Practitioners parameterise simplified or physically reduced heat-pump models from individual manufacturer datasheets [9,10], adopt national-aggregate hourly COP time series constructed from a few representative units [11], or draw per-product performance curves from certificate libraries such as hplib [12]. Reviews of domestic heat-pump performance show why the choice of bridge matters: the COP falls systematically with temperature lift, so two products with the same declared SCOP can differ materially in when and how much electricity they draw [13]. We are not aware of an existing route that converts a single declared seasonal value — the quantity practitioners actually hold at the design or EPC stage — into an hourly profile while guaranteeing that the profile reproduces the declared SCOP when integrated back over the rating conditions.

A further difficulty is evidential. A single datasheet describes one unit, while aggregate series suppress product-to-product variation entirely. Public certification databases offer a middle ground: large, quality-controlled populations of declared part-load performance. This population treatment of certification evidence was established for solar-thermal collectors with the Solar Keymark database [14], and the Heat Pump Keymark scheme [3] publishes the corresponding EN 14825 part-load test points for heat pumps; the validity of declared heat-pump efficiency data has also been examined independently [15]. The related research article [16] builds on this basis: it deduplicates the public air-to-water corpus and fits a mixed-effects population COP model whose fixed effects provide a defensible default COP(ΔT) shape when no product has been selected. Field trials, by contrast, consistently find in-use seasonal performance below rated values [17,18]; that evidence is deliberately kept outside the certified profile — the method anchors to the certified declared value, and any field-performance derating remains a separate, labelled adjustment (see the field-performance note below) rather than being folded into the conversion.

This method provides the missing certification-anchored bridge. It converts a declared SCOP into an hourly, capacity-aware COP profile in four steps: a heating curve maps each outdoor temperature to a lift; the population model returns the COP shape over that lift; an anchoring factor calibrates the profile so that the modelled system reproduces the declared SCOP over the rating bins; and a capacity-based split adds backup heating below the bivalent temperature using capacities already declared on the certificate. The method requires only standard EN 14825 certificate quantities, is independent of any particular simulation tool, and applies to certified air-to-water heat pumps with sufficient EN 14825 data, for climates with hourly outdoor-temperature data and an appropriate design temperature. It is therefore useful to building-energy modellers, to energy-system analysts who need time-resolved heat-pump operation, and to regulators setting climate-specific defaults. The fitted coefficients, an open-source Python implementation, and automated tests that reproduce the worked example are provided in a versioned deposit so that the method can be reproduced and embedded directly [2].

## Method details

### Inputs, usage modes and assumptions

The method has two usage modes. **Mode A — shape reconstruction (Steps 1–3)** produces an anchored hourly COP profile from the declared SCOP and climate data alone; because no explicit backup is added, the certification-condition backup and auxiliary-mode electricity embedded in the declared SCOP remain folded into the profile’s seasonal level, which then acts as an *effective* COP. This is the EPC/default-SCOP use case. **Mode B — capacity-aware system model (Steps 1–4)** additionally splits the load between heat pump and electric backup from declared capacities, and anchors so that backup electricity is counted exactly once (Step 3).

User-supplied:

• design supply-water temperature $T_{\text{supply},\text{design}}$ (e.g. 35 °C for underfloor, 55 °C for radiator systems);
• the declared seasonal COP $\text{SCO}P_{\text{user}}$ for that supply temperature and for a stated EN 14825 rating climate zone (from the product certificate or a tabulated default) — the anchoring bins in Step 3 are those of that rating zone;
• a climate definition that fixes the design outdoor temperature $T_{\text{design}}$ and either the EN 14825 bin hours $h_{j}$ or an hourly outdoor-temperature series;
• per application, the building heat load at each bin or hour $Q_{j}$ — the simulation’s net heat demand, or the EN 14825 part-load line scaled to the design load.

From the certificate (EN 14825), for Mode B:

• operating-limit temperature $T_{\text{OL}}$ (certificate parameter 005) and bivalent temperature $T_{\text{biv}}$ (004);
• the design heating load $P_{\text{design},h}$ (002) and the declared heat-pump heating capacities at the operating limit, $P_{\text{HP}}\left(T_{\text{OL}}\right)$ (018), and at the bivalent temperature, $P_{\text{HP}}\left(T_{\text{biv}}\right)$ (016).

Two assumptions define the scope: above $T_{\text{biv}}$ the selected heat pump is assumed to cover the building load (capacity-adequate sizing — the certificate’s discrete capacity points do not form a full capacity map), and the resistance backup operates at COP=1.

### Population COP model

The enabling component is a three-coefficient mixed-effects population model for the COP as a function of the temperature lift $\Delta T=T_{\text{supply}}-T_{\text{out}}$ (K), fitted to the deduplicated Keymark population [16]:

$$\text{COP}\left(\Delta T\right)=\beta_{2}+\beta_{0}\text{ln}\left(\Delta T\right)+\beta_{1}/\sqrt{\Delta T}$$

The fixed-effect coefficients (full per-product sets in the Zenodo deposit [2]) are:

• all-data variant, fitted to 53,551 test points from 3395 products across all three EN 14825 rating zones: $\beta_{2}=7.2602,\beta_{0}=-1.8528,\beta_{1}=18.0463$;
• cold-only variant, fitted to the 11,397 Colder-zone points from 979 products (21 of the 11,418 Colder-zone rows are dropped in fitting): $\beta_{2}=11.7252,\;\beta_{0}=-2.5819,\;\beta_{1}=6.6052$.

**Fit quality and out-of-sample error**. The headline metrics are those of the fixed effects alone, because a default user receives only the fixed effects: on the Colder-zone test set the cold-only fixed effects reach R² = 0.924 in-sample and R² = 0.922 out-of-sample under manufacturer-grouped 10-fold cross-validation (whole manufacturers held out; per-fold 0.921 ± 0.019), with out-of-sample MAE 0.37 and RMSE 0.55 COP units (manufacturer-cluster bootstrap 95% CIs 0.34–0.41 and 0.50–0.62) and near-zero overall bias (+0.004 COP). The all-data fixed effects evaluated on the same Colder set give R² = 0.903 with a systematic overprediction at low lift (+0.32 COP mean signed error for ΔT < 30 K, versus −0.04 for the cold-only fit). Error tables stratified by lift band, and the conditional (fixed-plus-random-effects) fit statistics, are given in the supplementary material.

**Fitted range and extrapolation**. The certification test points supporting the model span lifts of 10.9–83 K overall; the cold-only variant is supported over 10.9–77 K, where the 55 °C application class contributes points up to 77 K but the 35 °C class only up to 63 K. Within this range the functional form is monotonically decreasing, and it remains monotone outside it, so extrapolation cannot produce the non-physical COP upturn of polynomial fits; monotonicity controls only the *shape* of an extrapolation, however, and says nothing about its accuracy — predictions beyond the fitted lift range (or above 55 °C supply) should be treated as unvalidated extrapolations. The COP is floored at 1.0 throughout; the floor is a modelling convention for the hourly-to-seasonal level — the efficiency of direct electric resistance — not a physical bound on transient operation.

**Coefficient choice**. For a declared SCOP, choose the variant by its rating zone — the cold-only fit for Colder-zone declarations, the all-data fit otherwise; in anchored use the residual sensitivity is small (−0.27% annual electricity in the worked example below). When no declared value is available and the unanchored shape level itself is used, choose by the application climate (cold-only for Colder-zone applications). Before anchoring the two variants differ by 1.2% (Colder, 55 °C) to 3.4% (Average, 35 °C) in the bin-integrated seasonal shape factor, converging to within 0.5% above ΔT ≈ 45 K (the raw curves cross at ≈ 55 K); the full matrix is in the supplementary material.

**Between-product uncertainty of the population shape**. Products with identical declared SCOPs differ in compressor technology, refrigerant circuit, and control, and these differences appear in the certified test points as between-product spread around the population curve. Anchoring removes most of it: across the 979 cold-model products, the p10–p90 spread of the COP at fixed lift narrows from 19%/13%/11% of the median (raw curves) to 10%/3%/6% after each product’s curve is normalised to a common anchored seasonal level, at ΔT = 25/40/55 K respectively. At ΔT = 70 K, however, anchoring removes essentially none of the spread (≈21% residual): product shapes diverge at the cold extreme, which coincides with the edge of the fitted range. The residual spread after anchoring, together with the out-of-sample error tables above, is the uncertainty a user of the population default should carry; the tables are reproduced in the supplementary material. Standardised frosting test conditions and the EN 14825 cyclic-degradation allowance are embedded in the certified test points, so their average effect is inside the fitted curve; real dynamic cycling, humidity-dependent defrost beyond the test conditions, control logic and hydraulics are not (see Limitations).

### Step 1 — Heating curve and temperature lift

The supply-water temperature follows a linear heating curve from the design point $\left(T_{\text{design}},T_{\text{supply},\text{design}}\right)$ to the no-heating point (20 °C, 20 °C):

$$T_{\text{supply}}\left(T_{\text{out}}\right)=20+\frac{T_{\text{supply},\text{design}}-20}{T_{\text{design}}-20}\left(T_{\text{out}}-20\right)$$

Below the design temperature the supply is held at $T_{\text{supply},\text{design}}$ (the curve is clamped, so the lift keeps growing only through the falling outdoor temperature); above 20 °C it is held at 20 °C. The 20 °C pin is the zero-lift limit of a weather-compensation curve — supply equals room temperature at zero load — and is deliberately distinct from the 16 °C base of the EN 14825 part-load line, which reflects internal and solar gains. The temperature lift at each outdoor temperature (or hour) is $\Delta T\left(T_{\text{out}}\right)=T_{\text{supply}}\left(T_{\text{out}}\right)-T_{\text{out}}$.

This linear form is not merely a convenient default. For the Colder rating zone, the outlet-water temperatures at which the certificates declare their variable-outlet part-load points (49.2/42.5/35.0/30.8/26.7 °C at −15/−7/+2/+7/+12 °C for the 55 °C class, and correspondingly for the 35 °C class, with $T_{\text{supply},\text{design}}$ at the −22 °C operating-limit point) lie — to data rounding of 0.01 K — exactly on this line: the application curve coincides with the outlet-water conditions of the rating itself. The only residual convention is the extrapolation below the −15 °C test point; anchoring with the −15 °C outlet value held constant instead of continuing the line changes the anchoring factor by at most 0.55% (55 °C) and 0.18% (35 °C). Real installations may use non-linear compensation curves or different pins: substituting any curve in Step 1 is supported, and because Step 3 then re-anchors on the same curve, re-anchoring strongly reduces the annual sensitivity to the curve choice in the tested cases — in the worked example, replacing the line by radiator-exponent curves (n = 1.1–1.5) changes annual electricity by at most +0.23%, and moving the pin to 18 °C or 22 °C by −0.31%/+0.24%. Changing the curve *without* re-running Step 3 changes annual electricity by up to ≈ +6.5% at n = 1.3 (≈ +10% at n = 1.5): the anchoring convention, not the curve itself, carries the seasonal level.

### Step 2 — Population COP shape

Evaluate the population model at each lift, floored at 1.0:

$$\text{CO}P_{\text{pop}}\left(\Delta T\right)=\text{max}\left(1,\beta_{2}+\beta_{0}\text{ln}\Delta T+\beta_{1}/\sqrt{\Delta T}\right)$$

This is the *shape* of the COP curve — its variation with temperature — not the absolute level of a specific unit.

### Step 3 — Seasonal anchoring to the declared SCOP

**What the declared value contains**. EN 14825 defines a ladder of seasonal metrics: $\text{SCO}P_{\text{net}}$ (active-mode efficiency of the heat pump alone, excluding the supplementary heater), $\text{SCO}P_{\text{on}}$ (active mode including the electric supplementary heater at COP = 1), and the overall SCOP, which additionally includes the off-mode, thermostat-off, standby and crankcase-heater consumptions. The SCOP declared on a Keymark certificate is this overall, auxiliary-inclusive quantity: recalculating it from each certificate’s own test points across 26,478 configurations reproduces the declared values with a Colder-zone mean deviation of −0.9% (see Method validation). The declared value therefore already contains the certification-condition backup and auxiliary electricity of the rated unit. Across the 5503 valid Colder-zone air-to-water configurations, the certification-condition backup-electricity share embedded in the declared SCOP has a median of 3.0% and a 90th percentile of 16.5% — largest for units whose declared operating limit lies above the zone design temperature — so how that share is handled is material at population level, and the two usage modes handle it explicitly.

**Anchoring integral**. The seasonal shape factor is computed over the EN 14825 bins of the declared SCOP’s rating zone, with the *same* heating curve as in the application (Step 1) — the anchoring convention of this method — where the part-load ratio is $p_{l}\left(T\right)=\left(T-16\right)/\left(T_{\text{design}}-16\right)$ for *T* < 16 °C and 0 otherwise, and the reference bin loads are $Q_{j}=p_{l}\left(T_{j}\right)\;P_{\text{design},h}$:

$$\text{SCO}P_{\text{shape}}=\frac{\sum_{j}h_{j}\;p_{l,j}}{\sum_{j}h_{j}\;p_{l,j}/\text{CO}P_{\text{pop}}\left(j\right)}$$

**Mode A (shape reconstruction)**. The anchoring factor is $\alpha =\text{SCO}P_{\text{user}}/\text{SCO}P_{\text{shape}}$, and the anchored hourly COP is

$$\text{CO}P_{\text{user}}\left(\Delta T\right)=\text{max}\left(1,\;\alpha \;\text{CO}P_{\text{pop}}\left(\Delta T\right)\right)$$

The anchored profile then reproduces $\text{SCO}P_{\text{user}}$ exactly when integrated back over the rating bins. Because the target is the auxiliary-inclusive declared value, the certification backup and auxiliary consumptions are folded into the profile’s level as a uniform derate — appropriate for Mode A, where no explicit backup is added downstream.

**Mode B (capacity-aware)**. When Step 4 adds explicit backup, anchoring the heat-pump share to the backup-inclusive declared value would count certification backup twice. Mode B therefore anchors the *full modelled system* over the rating bins — heat-pump share plus backup at COP = 1 — to the declared value:

$$\sum_{j}h_{j}\left[\frac{Q_{\text{HP},j}}{\text{max}\left(1,\alpha_{\text{bu}}\text{CO}P_{\text{pop}}\left(j\right)\right)}+Q_{\text{bu},j}\right]=\frac{\sum_{j}h_{j}Q_{j}}{\text{SCO}P_{\text{user}}}$$

with the bin-wise capacity split $Q_{\text{HP},j}$, $Q_{\text{bu},j}$ of Step 4 evaluated on the reference bin loads. When the COP floor is inactive over the anchoring bins this has the closed form $\alpha_{\text{bu}}=\sum_{j}h_{j}Q_{\text{HP},j}/\text{CO}P_{\text{pop}}\left(j\right)\;/\;\left(\sum_{j}h_{j}Q_{j}/\text{SCO}P_{\text{user}}-\sum_{j}h_{j}Q_{\text{bu},j}\right)$; when the floor binds (low declared SCOPs), α is obtained by a one-dimensional monotone numerical solve of the seasonal target to the specified tolerance — the reference implementation uses bisection and reproduces the target to machine precision in the shipped tests. The Mode-B equation reduces exactly to Mode A when the certificate shows no capacity shortfall ($Q_{\text{bu},j}=0$). Backup electricity is thus counted exactly once in each mode: implicitly in the level (Mode A) or explicitly in Step 4 (Mode B); auxiliary-mode consumptions are implicit in the anchor in both modes and never added explicitly.

Anchoring is an **internal-consistency guarantee of the conversion, not evidence of predictive validity**: it verifies the calibration arithmetic and ties the profile to the certificate, but it cannot show that the hourly *distribution* of the COP around the seasonal level is right. That evidence comes from the out-of-sample shape validation and the external evidence reported under Method validation. As a certification-reference check, replacing the application curve in the anchoring integral by the rating outlet schedule with its −15 °C extrapolation convention changes α by at most 0.55% (see Step 1).

### Step 4 — Capacity-based backup split and system electricity

Below the bivalent temperature the declared heat-pump capacity cannot meet the full building load. The available capacity is interpolated linearly between the two declared capacity points,

$$P_{\text{HP},\text{lin}}\left(T_{\text{out}}\right)=P_{\text{HP}}\left(T_{\text{OL}}\right)+\left(P_{\text{HP}}\left(T_{\text{biv}}\right)-P_{\text{HP}}\left(T_{\text{OL}}\right)\right)\;\frac{T_{\text{out}}-T_{\text{OL}}}{T_{\text{biv}}-T_{\text{OL}}},\;T_{\text{OL}}\leq T_{\text{out}}\leq T_{\text{biv}}$$

and the load is split against the actual bin or hourly load: $Q_{\text{HP},j}=\text{min}\left(Q_{j},P_{\text{HP},\text{lin}}\left(T_{j}\right)\;\right)$ per unit time and $Q_{\text{bu},j}=Q_{j}-Q_{\text{HP},j}$. Above $T_{\text{biv}}$ the heat pump covers the load (capacity-adequate sizing assumption); below $T_{\text{OL}}$ the heat pump is off and the load is met entirely by backup electric resistance. Linear interpolation between declared test-point quantities mirrors the interpolation convention of the EN 14825 calculation itself and uses only certificate fields (codes 016/018); it is an engineering approximation consistent with the certified data structure, not a prescribed physical law — units whose capacity varies strongly non-linearly between the two points, fixed-speed or cascade units, and certificates where $T_{\text{biv}}\approx T_{\text{OL}}$ (the interpolation collapses toward a step) are represented only approximately. The derived capacity share $\Phi_{j}=Q_{\text{HP},j}/Q_{j}$ replaces the fixed-share ramp of earlier formulations, and responds to the actual load within the TOL–Tbiv window, where the backup share grows automatically under undersizing; above Tbiv the capacity-adequate sizing assumption applies, so a general undersizing treatment requires additional certified capacity points (see Limitations). For each bin or hour j, the electricity input combines the heat-pump share (at $\text{CO}P_{\text{user}}$, Mode-B anchored) and the backup share (at COP = 1):

$$E_{j}=\frac{Q_{\text{HP},j}}{\text{CO}P_{\text{user}}\left(T_{j}\right)}+Q_{\text{bu},j},\;\text{SCO}P_{\text{sys}}=\frac{\sum_{j}Q_{j}}{\sum_{j}E_{j}}$$

Summing $E_{j}$ over the year yields the seasonal electricity use and the realised system seasonal coefficient $\text{SCO}P_{\text{sys}}$. In the worked example the split governs 166 blend hours (2.2% of heating hours) and 1.7% of delivered heat, so plausible deviations from linear capacity variation are second-order there; the share grows in colder climates, for wider $T_{\text{OL}}$–$T_{\text{biv}}$ intervals, and under undersizing. A reference Python implementation (NumPy-only, standalone) that performs all four steps in both modes is provided in the Zenodo deposit [2].

### Output variables

The method returns, per bin or hour: the temperature lift ΔT, the anchored heat-pump COP $\text{CO}P_{\text{user}}$, the heat-pump and backup load shares, and the electricity input $E_{j}$; and, per application: the seasonal electricity use and the realised $\text{SCO}P_{\text{sys}}$. $\text{SCO}P_{\text{sys}}$ is an author-defined simulation output — the seasonal heat-to-electricity ratio of the modelled system on the application climate and load. It is not an EN 14825 metric (it contains no explicit auxiliary-mode terms; those ride implicitly in the anchor) and not a field SPF; on the rating bins themselves the Mode-B system value equals the declared SCOP by construction, while on a different application climate it legitimately differs.

### Field-performance adjustment (scope note)

Field trials consistently measure in-use seasonal performance below rated values [17,18]. The conversion deliberately anchors to the *certified* value; a field adjustment is applied, when wanted, as a separate labelled step **before** Step 3: replace $\text{SCO}P_{\text{user}}$ by $f_{\text{field}}\cdot \text{SCO}P_{\text{user}}$ with a user-chosen derating factor. In Mode A the anchoring factor is linear in the target, so electricity scales as $1/f_{\text{field}}$; in Mode B the explicit backup term is unaffected and α_bu_ responds slightly non-linearly: at $f_{\text{field}}=0.85$ the realised increase (+17.0%) falls just below the naive (1/0.85−1=+17.6%). A derate changes only the seasonal *level*; the population temperature *shape* is untouched. Reported field-gap ranges (typically 10–30% depending on system boundary, climate, hot-water inclusion and controls [17,18]) are context for choosing $f_{\text{field}}$, not a prescribed universal factor.

### Domestic hot water (scope note)

The core method applies to EN 14825 space-heating SCOP. Domestic hot water is certified under EN 16147 [19] as a single-point COP at a fixed ambient temperature rather than as a seasonal bin-integrated value, so it lies outside the validated core of this method. An optional extension can anchor the population COP shape to the EN 16147 point and integrate it over an hourly draw profile, as demonstrated for one unit in the related research article [16], where the seasonal DHW COP comes out about 4% above the single declared point; because it rests on a single declared point and a modelled draw profile, any such DHW result should be treated as indicative rather than as a validated core output.

**Pseudocode**.

**Figure:**
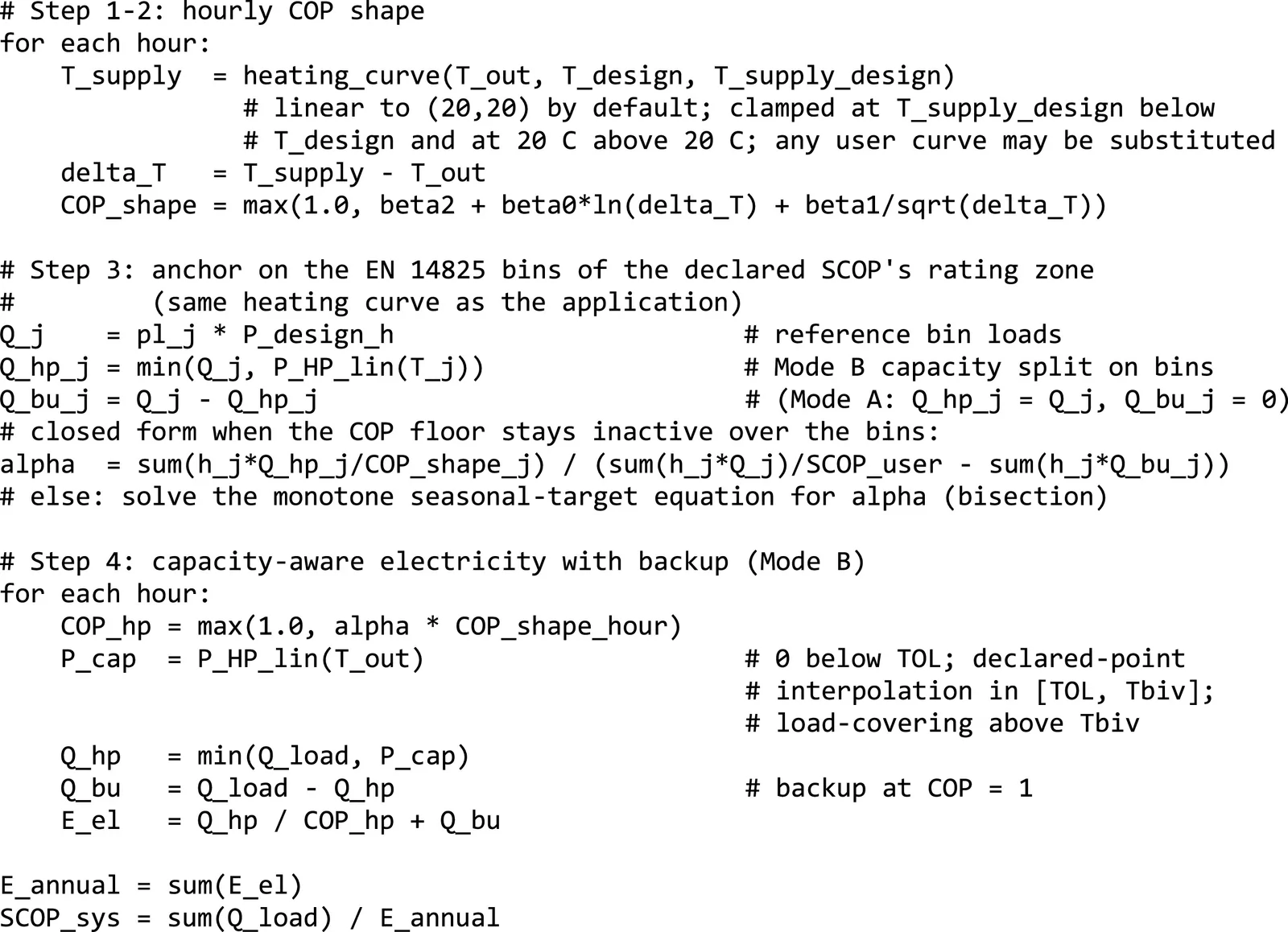

### Two-stage worked example

The example uses a certified 9–12 kW air-to-water unit whose declared operating-limit and bivalent temperatures sit at the cold-climate population medians [16], at 55 °C supply, with the cold-only coefficients, in Mode B. It is deliberately presented in two stages, separating what the certificate fixes from what the application climate adds. **Stage A** decomposes the declared SCOP over its own EN 14825 Colder rating bins: this is where the anchoring is exact. **Stage B** applies the anchored profile, unchanged, to the hourly Estonian test reference year (TRY) and the hourly space-heating load profile of a reference single-family-house archetype (deposited with the code) — the climate-adaptation step, where the realised system value legitimately differs from the declared one. All values are reproduced by the Python implementation and the automated tests in the Zenodo deposit [2]; table values are rounded. Heating hours are defined by non-zero load in the archetype profile (7518 h; 7242 of them below 16 °C outdoor temperature — the remainder are near-zero-load mild hours carrying 0.16% of annual demand).

Unit and application inputs:

**Table:** 

Quantity	Value
Declared $\text{SCO}P_{\text{user}}$ (Colder, 55 °C)	3.26
$T_{\text{supply},\text{design}}$	55 °C
$T_{\text{design}}$	−22 °C
$T_{\text{OL}}$; $T_{\text{biv}}$	−22 °C; −15 °C
$P_{\text{design},h}$	11.0 kW
$P_{\text{HP}}\left(T_{\text{OL}}\right)$; $P_{\text{HP}}\left(T_{\text{biv}}\right)$	8.0 kW; 8.97 kW
Application climate / load	Estonian TRY, hourly; 33,000 kWh/yr archetype load profile

Stage A — declared-SCOP decomposition on the EN 14825 Colder bins:

**Table:** 

Quantity	Value
$\text{SCO}P_{\text{shape}}$ over the rating bins	3.128
$\alpha_{\text{bu}}$ (Mode B, closed form; floor inactive)	1.049
Full-system bin integral (HP + backup)	3.2600 = declared (|error| < 10⁻¹³)
Heat-pump-only bin integral	3.280
Certification-bin backup electricity share	1.7%

Stage B — application to the Estonian TRY (same anchored profile):

**Table:** 

Quantity	Value
Annual electricity	9938 kWh
Realised $\text{SCO}P_{\text{sys}}$	3.32
Backup: share of heat / of electricity	1.7% / 5.5%
Blend hours; backup-only hours	166; 1
Minimum anchored COP in heat-pump operation (at ΔT = 76.6 K)	1.34
Hours with the COP floor active	0

The Stage-B $\text{SCO}P_{\text{sys}}$ (3.32) lies *above* the declared 3.26 because the Estonian TRY weights mild lifts more heavily than the Colder rating-bin template; exactness belongs to Stage A by construction, and Stage B reports what the certificate-anchored unit does in the application climate. The design point (−22 °C at 55 °C supply, ΔT = 77 K) sits at the fitted-range boundary of the cold-only model; the single coldest TRY hour (−22.1 °C) lies below the operating limit and is met entirely by backup, so the heat pump never operates above the 77 K fitted maximum — the minimum COP in actual heat-pump operation is 1.34 at ΔT = 76.6 K (the 1.32 curve value at the 77.1 K backup-only hour is never used). The floor never binds in this example; over the anchoring bins the floor first activates at declared SCOPs below ≈ 2.5, where the numerical solve of Step 3 replaces the closed form (reproduction of the seasonal target then remains exact to solver tolerance; see the deposited tests). Repeating Stage B with the all-data coefficients changes annual electricity by −0.27%; sensitivity brackets for the heating-curve shape and pin are given in Step 1, and the full sensitivity and threshold tables are in the supplementary material.

### Method validation

The evidence is organised in four tiers — computational verification of the implementation, out-of-sample validation of the population shape, external evidence (an external laboratory hold-out and an implementation benchmark), and a system-integration consistency check — so that internal-consistency properties are not mistaken for predictive evidence. Unless otherwise stated, the numerical validation results below are reproduced by the scripts, data and automated tests archived in the deposit [2] and are reported in full in the supplementary material; the WPZ, hplib and plant-check values use the sources described there.

**Tier 1 — Computational verification**.

**Table:** 

Check	Result
EN 14825 Annex H worked example (bin calculation underlying Step 3; auxiliary powers zero in Annex H, so the check exercises $\text{SCO}P_{\text{on}}$)	3.60 vs reference 3.61 (0.3%)
Batch recalculation of the declared SCOP from each certificate’s own test points (26,478 configurations; auxiliary-inclusive overall SCOP)	87.0% within ±5%, 96.9% within ±10%; Colder-zone mean −0.9%, median −0.8%
Anchoring identity (Stage A): closed form where the COP floor is inactive; monotone numerical solve where it binds	seasonal target reproduced to |error| < 10⁻¹³ across all tested template/coefficient combinations; both paths covered by shipped automated tests

**Tier 2 — Out-of-sample shape validation (unseen certified products)**. Manufacturer-grouped 10-fold cross-validation of the fixed effects (whole manufacturers held out, so every evaluation product comes from a manufacturer absent from training): cold-only R² 0.922 out-of-sample vs 0.924 in-sample (gap +0.003; per-fold 0.921 ± 0.019); leave-one-manufacturer-out over the 55 manufacturers with ≥5 Colder-zone points gives median held-out R² 0.933 (IQR 0.905–0.958), with 91% of manufacturers above 0.80. Out-of-sample MAE/RMSE 0.37/0.55 COP with near-zero overall bias; stratified error tables in the supplementary material.

As a temporal hold-out, the same frozen fixed effects were additionally evaluated, without refitting, on the air-to-water models first observed in a 20 August 2026 Keymark snapshot and absent from the 18 March 2026 training corpus after the same deduplication and identity screens: 66 snapshot-unseen models with Colder-zone test points, from 16 manufacturers, with 725 points inside the fitted lift range. Temporal MAE and RMSE (0.37 / 0.56 COP) match the manufacturer-grouped cross-validation values (ratios 1.00 / 1.02), while the newest products run systematically above the frozen population curve (mean signed error −0.25 COP, concentrated below ΔT = 30 K): the frozen default is conservative for post-snapshot products, and in anchored use the selected product’s declared SCOP attenuates much of this level shift. The cohort cascade and stratified results are reported in the supplementary material; no model component was refitted, retuned or selected using the temporal results. This tests temporal generalisation within the certification domain; it does not constitute validation of non-certified or field-installed systems.

**Tier 3 — External evidence**.

**External laboratory hold-out (WPZ** [20]). Without refitting, the frozen cold-only fixed effects were evaluated against 1021 in-range COP measurements of 102 air-to-water units published by the WPZ Buchs heat-pump test centre (49 manufacturers, 39 of them absent from the training corpus; every unit screened against the frozen corpus by manufacturer, model alias and measurement signature, two identity hits excluded). The overall MAE was 0.45 COP (manufacturer-cluster 95% CI 0.39–0.52) with negligible mean bias (−0.05 COP, CI −0.21 to +0.08); for the 656 EN 14511 full-load points, MAE was 0.35 COP and bias +0.04. Larger negative residuals were concentrated in low-lift EN 14825 part-load measurements of newer units (bias −0.22), consistent with the level shift observed in the temporal hold-out. This is an external laboratory check under standardised test conditions, not field validation (Supplement S-K).

**Implementation benchmark (hplib** [12]). Against the independently developed hplib library, which parameterises 9314 air/water units from public Heat Pump Keymark certification data: at mid-lift standard points such as A − 7/W35 and A + 2/W55 the population curve lies inside the hplib interquartile range, with divergence growing toward the extreme corners (hplib’s per-product lines hit their own floor at A − 15/W55). In a building-level head-to-head on the same load and climate, the population default predicts annual heat-pump electricity within +2.5% of the hplib median unit, and the population percentile bands bracket it. This is an external implementation benchmark — an alternative, per-product parameterisation within broadly the same Keymark certification-data domain — not an independent-data validation.

**Tier 4 — System-integration consistency check (IDA ICE)**. Reproducing a dynamic IDA ICE on/off plant model of an Estonian single-family house on the same delivered heat: space heating +0.1%, domestic hot water −1.8%, total −0.9% at the reference plant-control setting; across seven plant-control settings the total spans approximately ±10%, driven mainly by the DHW electric top-up control. This is a single-case model-consistency check for the tested house — not a general validation across building types, climates, control strategies, or sizing ratios.

## Limitations

The method is intended for certified air-to-water heat pumps with sufficient EN 14825 space-heating data. It converts declared certificate performance into a simulation-ready COP profile, but it is not a field-performance model and does not account for installation quality, hydraulic design, real dynamic cycling and humidity-dependent defrost beyond the standardised test conditions and cyclic-degradation allowance embedded in the certificate data, user behaviour, or control faults; a field derate is available only as the separate labelled adjustment described above. The population shape is supported over lifts of ≈ 11–77 K (cold-only; 35 °C applications to 63 K) — beyond that range, and above 55 °C supply, predictions are unvalidated extrapolations of a monotone form, and between-product shape divergence at the cold extreme is not reduced by anchoring (residual p10–p90 spread ≈ 21% at ΔT = 70 K). The temporal hold-out cohort also contained 91 Colder-zone points (11.2%) above the frozen 77 K fitted limit, reaching 80 K; these were excluded from the headline temporal errors and reported separately. Below the certified operating-limit temperature, performance is represented by explicit electric backup rather than extrapolated compressor operation. The Step-4 capacity split interpolates between two declared capacity points and assumes capacity-adequate sizing above the bivalent temperature; strongly non-linear capacity characteristics, fixed-speed and cascade units, and certificates with $T_{\text{biv}}\approx T_{\text{OL}}$ are represented only approximately. Auxiliary-mode consumptions are carried implicitly in the anchored level, not as explicit terms. Domestic hot water performance is less resolved because EN 16147 provides a single-point metric rather than a seasonal bin-integrated SCOP; any DHW extension should therefore be treated as indicative. Laboratory or declaration bias in the source certificates propagates into the resulting profile unless corrected externally. Ground-source, exhaust-air, and air-to-air units differ in both physics and certified data structure and are out of scope.

## Related research article

K.-V. Võsa, A. Mikola, J. Kurnitski, R. Simson, Certified heat-pump performance assumptions in building-energy simulation: representative-product choice outweighs COP time resolution, submitted to Energy and Buildings (2026).

## Ethics statements

Not applicable. This work did not involve human subjects, animal experiments, or data collected from social media platforms.

## CRediT authorship contribution statement

**Karl-Villem Võsa:** Conceptualization, Methodology, Software, Data curation, Formal analysis, Writing – original draft. **Alo Mikola:** Investigation, Validation, Writing – review & editing. **Jarek Kurnitski:** Supervision, Funding acquisition, Writing – review & editing. **Raimo Simson:** Supervision, Methodology, Writing – review & editing.

## Declaration of competing interest

The authors declare that they have no known competing financial interests or personal relationships that could have appeared to influence the work reported in this paper.

## Data Availability

CC BY 4.0 https://doi.org/10.5281/zenodo.22029915.

## Appendix Supplementary materials

A supplementary file accompanies this article with: the model fit and random-effects statistics (including the conditional fit metrics), the manufacturer-grouped cross-validation protocol and stratified out-of-sample error tables, the temporal hold-out on post-snapshot Keymark additions, the external laboratory hold-out against WPZ test-centre measurements (cohort, screening and error tables), the EN 14825 batch-recalculation and Annex H details, the full Stage-A bin decomposition table, the heating-curve and rating-schedule sensitivity tables, the coefficient-variant matrix, the anchored between-product spread table, floor-activation thresholds, and compact hplib and IDA ICE benchmark tables. The standalone Python implementation, the fitted coefficients, the worked-example inputs and expected outputs, the validation scripts, and automated tests reproducing every printed example value are archived in the versioned Zenodo deposit (https://doi.org/10.5281/zenodo.22029915, CC BY 4.0). Image, application 1
