# Supplementary material for: Four-step conversion of a declared seasonal coefficient of performance into hourly, capacity-aware coefficient-of-performance profiles for certified air-to-water heat pumps
Source: MethodsX. 2026 Aug 28;17:104120. doi: 10.1016/j.mex.2026.104120 (PMC13571556; doi:10.1016/j.mex.2026.104120)
Supplement: Supplementary file 1 [file mmc1.docx]

# Supplementary material

Supplementary material to: **Four-step conversion of a declared seasonal coefficient of performance into hourly, capacity-aware coefficient-of-performance profiles for certified air-to-water heat pumps**

*Karl-Villem Võsa, Alo Mikola, Jarek Kurnitski, Raimo Simson*

Unless otherwise stated, the numbers in this supplement are generated by the scripts and data archived in the Zenodo deposit (<https://doi.org/10.5281/zenodo.22029915>; cite the release version matching this article); the hplib and IDA ICE benchmark values derive from the sources described in Section S-H and the WPZ laboratory values from the source described in Section S-K. Section pointers to the generating scripts are given in each caption.

## S-A. Population-model provenance and fit statistics

The mixed-effects model $COP=\beta_{2}+\beta_{0}ln\Delta T+\beta_{1}/\sqrt{\Delta T}$ is fitted with a per-product random effect on all three coefficients, on the deduplicated Heat Pump Keymark air-to-water corpus.

**Table S-A1. Model variants and fit statistics.** “Marginal” = fixed effects only (what a default user receives, COP floored at 1.0, evaluated on the Colder-zone test set); “conditional” = fixed plus per-product random effects (not available to a default user; reported for completeness).

| Variant | Training points | Products | β₂ | β₀ | β₁ | Marginal R² (Colder) | Conditional R² | RMSE (cond.) |
| --- | --- | --- | --- | --- | --- | --- | --- | --- |
| All-data (3 zones) | 53,551 | 3,395 | 7.2602 | −1.8528 | 18.0463 | 0.903 | 0.956 | 0.408 |
| Cold-only (Colder zone) | 11,397 | 979 | 11.7252 | −2.5819 | 6.6052 | 0.924 | 0.973 | 0.323 |

**Cross-evaluation on the Colder-zone set (n = 11,418):** the all-data fixed effects carry a mean bias of +0.178 COP (overprediction), concentrated at low lift (+6.5% mean relative error at ΔT < 30 K); the cold-only fixed effects are essentially unbiased (+0.001 COP). Empirical per-product random-effect spreads (BLUP-based, hence slightly shrunken relative to the true population variance): cold-only SD(β₀) = 0.313, SD(β₁) = 1.509, SD(β₂) = 1.087 with near-degenerate correlations (|r| = 0.97–0.99) — product curves vary mainly along a single level-like direction, which is why a scalar anchoring factor removes most between-product variance (Section S-G).

**Training-data coverage.** Test-point lifts span ΔT = 10.9–83.0 K overall (p5–p95: 12–65 K). Cold-only subset: 10.9–77 K, where the 55 °C application class contributes points to 77 K and the 35 °C class to 63 K; outdoor temperatures −28 to +12 °C; supply temperatures 22.9–55.0 °C. The Colder 55 °C design point (−22 °C, ΔT = 77 K) sits at the upper edge of the fitted range.

**Table S-A2. Coefficient-variant divergence before anchoring: bin-integrated seasonal shape factor** $\mathrm{SCOP}_{\mathrm{shape}}$ **(all-data / cold-only; relative difference).** The unfloored raw curves cross at ΔT ≈ 55 K and agree within ±0.2% over 50–70 K; the all-data curve reads higher at low lift (+5.1% at 20 K, +2.5% at 30 K), which drives the divergence for mild templates and low supply temperatures. After anchoring to a declared SCOP the residual effect on the worked example’s annual electricity is −0.27%.

| Bin template | 55 °C | 35 °C |
| --- | --- | --- |
| Average (T_design = −10 °C) | 3.412 / 3.366 (+1.4%) | 4.741 / 4.586 (+3.4%) |
| Colder (T_design = −22 °C) | 3.164 / 3.128 (+1.2%) | 4.249 / 4.141 (+2.6%) |

*Source:* models/ and validation/anchored_spread.py, validation/oos_error_tables.py in the data deposit.

## S-B. Out-of-sample validation protocol and error tables

**Protocol.** Manufacturer-grouped 10-fold cross-validation: whole manufacturers are assigned to folds deterministically (largest first, into the currently lightest fold), the three-coefficient fixed-effects model is refitted on each training fold, predictions are floored at COP = 1.0, and evaluation uses only points from held-out manufacturers — every evaluated product comes from a manufacturer absent from training. Cold-only protocol: fit and evaluate within the Colder zone (55 manufacturers). All-data protocol: fit on all zones minus the held-out manufacturers, evaluate on the Colder zone. Leave-one-manufacturer-out (LOMO) repeats the procedure per manufacturer with ≥5 Colder points.

**Headline:** cold-only pooled out-of-sample R² = 0.922 vs in-sample 0.924 (per-fold 0.921 ± 0.019; generalisation gap +0.003); all-data 0.903 vs 0.905. LOMO: median held-out R² 0.933, IQR 0.905–0.958, 91% of manufacturers above 0.80, minimum 0.577.

**Table S-B1. Out-of-sample error tables by lift band (signed error = predicted − observed; relative error = signed/observed).** Empirical error distributions for unseen manufacturers — not calibrated prediction intervals (coverage was not calibrated).

Cold-only protocol (fit Colder, evaluate Colder):

| ΔT band | n | MAE | RMSE | Mean bias | Median bias | rel. p5 | p25 | p50 | p75 | p95 |
| --- | --- | --- | --- | --- | --- | --- | --- | --- | --- | --- |
| < 30 K | 4,824 | 0.571 | 0.761 | −0.045 | −0.070 | −17.8% | −7.6% | −1.3% | +7.3% | +22.9% |
| 30–55 K | 4,298 | 0.221 | 0.314 | +0.095 | +0.079 | −10.0% | −2.3% | +2.7% | +8.6% | +22.8% |
| > 55 K | 2,296 | 0.224 | 0.318 | −0.066 | −0.065 | −27.3% | −11.4% | −3.7% | +6.5% | +22.8% |
| Overall | 11,418 | 0.369 | 0.550 | +0.004 | +0.015 | −18.5% | −6.4% | +0.4% | +7.8% | +22.9% |

All-data protocol (fit all zones, evaluate Colder):

| ΔT band | n | MAE | RMSE | Mean bias | Median bias | rel. p5 | p25 | p50 | p75 | p95 |
| --- | --- | --- | --- | --- | --- | --- | --- | --- | --- | --- |
| < 30 K | 4,824 | 0.640 | 0.863 | +0.317 | +0.259 | −13.3% | −3.2% | +4.2% | +13.2% | +30.1% |
| 30–55 K | 4,298 | 0.234 | 0.328 | +0.135 | +0.123 | −8.8% | −1.0% | +4.1% | +10.0% | +24.4% |
| > 55 K | 2,296 | 0.213 | 0.306 | −0.024 | −0.030 | −24.1% | −8.6% | −1.6% | +8.5% | +25.5% |
| Overall | 11,418 | 0.401 | 0.611 | +0.180 | +0.107 | −14.5% | −3.5% | +3.4% | +11.2% | +27.8% |

**Manufacturer-cluster bootstrap 95% CIs** (500 replicates, manufacturers resampled with replacement): cold-only MAE 0.369 [0.341, 0.409], RMSE 0.550 [0.498, 0.623]; all-data MAE 0.401 [0.353, 0.440], RMSE 0.611 [0.534, 0.678]. The CIs overlap, so the cold-only advantage in MAE/RMSE is directional at cluster level; the low-lift bias difference (+0.317 vs −0.045 COP) is the substantive contrast.

*Source:* validation/oos_error_tables.py in the data deposit.

**Temporal hold-out on post-snapshot Keymark additions.** The fixed effects, preprocessing, deduplication and identity screens frozen at the 18 March 2026 training snapshot were evaluated, without refitting, on the certificate records first observed in a fresh Heat Pump Keymark snapshot retrieved on 20 August 2026. Cohort cascade: 191 new certificate records carrying 481 model entries → 478 after excluding identity matches to training models → 346 after excluding records whose frozen OEM measurement signature already occurs in training (re-badges) → 342 after certificate-lineage renewals (partial registry coverage) → 25 borderline same-manufacturer name-variants (year-edition and generation-suffix records) excluded from the primary cohort by pre-committed identity rules resolved before residuals were computed (none carries Colder-zone points, so this exclusion does not change the primary numbers) → 187 unique measurement signatures, of which 164 are air-to-water and **66 models (16 manufacturers) carry Colder-zone test points**: 816 Colder points, 725 inside the fitted lift range (10.9–77 K), 91 outside (up to 80 K). All 66 models have certificate issue dates after the cutoff.

**Table S-B2. Temporal hold-out errors (frozen cold-only fixed effects; point-weighted; signed error = predicted − observed).**

| Set | n | R² | MAE | RMSE | Mean bias | Median bias |
| --- | --- | --- | --- | --- | --- | --- |
| In-range combined | 725 | 0.905 | 0.371 | 0.559 | −0.253 | −0.171 |
| ΔT < 30 K | 264 | 0.292 | 0.614 | 0.841 | −0.490 | −0.396 |
| ΔT 30–55 K | 281 | 0.780 | 0.235 | 0.304 | −0.070 | −0.083 |
| ΔT > 55 K | 180 | −0.113 | 0.226 | 0.280 | −0.189 | −0.168 |
| Tier N-A (unseen manufacturers; 12 models, 6 mfrs) | 138 | 0.846 | 0.495 | 0.804 | −0.356 | −0.133 |
| Tier N-B (unseen models, known manufacturers) | 587 | 0.924 | 0.342 | 0.484 | −0.228 | −0.200 |
| Out-of-range only (sensitivity) | 91 | 0.911 | 0.569 | 0.879 | −0.410 | −0.330 |
| All Colder points (sensitivity) | 816 | 0.918 | 0.393 | 0.603 | −0.270 | −0.189 |

Model-level distribution (66 models): MAE median 0.31, IQR 0.25–0.38; bias median −0.21, IQR −0.32…−0.05. Manufacturer-cluster bootstrap (500 replicates): MAE 95% CI [0.29, 0.51], RMSE [0.39, 0.78]. Temporal-to-CV ratios: MAE 1.00, RMSE 1.02 — the error magnitudes match the cross-validation values, but the mean signed bias (−0.25 COP, largest below ΔT = 30 K) exceeds the pre-committed ±0.10-COP consistency wording gate: the newest certified products systematically outperform the frozen population curve at mild lifts, i.e. the frozen default is conservative for post-snapshot products. Consistently, the all-data fixed effects — which read higher at low lift — sit closer to the temporal cohort (bias −0.130, MAE 0.328). In anchored use the selected product’s declared SCOP attenuates much of the level shift (the reconstruction diagnostic below retains a median bias of −0.13 COP after SCOP conditioning); for unanchored shape-level use the conservatism is a stated limitation, not corrected by refitting.

Secondary checks: a reconstruction diagnostic conditional on the declared SCOP (Mode A boundary, chosen before the run) over the 130 cohort configurations with a declared Colder SCOP gives product-level MAE median 0.30 (IQR 0.22–0.41) and bias median −0.13 — reported as a use-case diagnostic, not independent validation, since the declared SCOP derives from the same certificate evidence. Recalculating each temporal certificate’s declared SCOP with the unchanged EN 14825 pipeline (130 valid recalculations) reproduces the declared values to mean −0.5% (SD 0.5%) — a data-integrity check confirming the fresh records are parsed consistently with the training snapshot. This exercise evaluates temporal generalisation within the Heat Pump Keymark certification domain only; it does not validate non-certified products or installed-system field performance. The model form, coefficients, preprocessing rules, deduplication logic, screening rules and error metrics were frozen before the later snapshot was evaluated; no model component was refitted, retuned or selected using the temporal hold-out results.

*Source:* validation/temporal_holdout.py, cohort and error files in scop_validation/ (temporal_*.csv), and code/test_temporal_holdout.py in the data deposit.

## S-C. EN 14825 computational verification

**Metric ladder.** $\mathrm{SCOP}_{\mathrm{net}}$: active-mode efficiency of the heat pump alone (supplementary heater excluded). $\mathrm{SCOP}_{\mathrm{on}}$: active mode including the electric supplementary heater at COP = 1. Overall SCOP: adds the off-mode, thermostat-off, standby and crankcase-heater energies (certificate parameters 023–026). The Keymark-declared SCOP (certificate parameter 003) is the overall, auxiliary-inclusive quantity.

**Annex H check.** Our EN 14825 bin implementation reproduces the Annex H worked example: $\mathrm{SCOP}_{\mathrm{on}}$ 3.5976 → 3.60 vs reference 3.61 (0.3%, attributable to extrapolation at the 13–15 °C bins), $\mathrm{SCOP}_{\mathrm{net}}$ 3.6648 → 3.66. Auxiliary powers are zero in the Annex H example, so this check exercises $\mathrm{SCOP}_{\mathrm{on}}$.

**Batch recalculation vs declared values.** Recalculating the auxiliary-inclusive overall SCOP from each certificate’s own test points (each certificate’s declared auxiliary powers and degradation coefficients included): of 26,904 attempted configuration × climate rows, 26,478 (98.4%) had sufficient data. Deviations from declared values: all-zone mean −2.15%, median −1.31%, SD 3.20%; Colder zone mean −0.91%, median −0.77%, SD 2.74%; 87.0% of rows within ±5%, 96.9% within ±10%. This verifies the implementation at population scale and corroborates its consistency with the declared overall-SCOP interpretation.

**Anchoring identity.** The Step-3 solve reproduces the seasonal target to |error| < 10⁻¹³ in all tested template/coefficient combinations, by closed form where the COP floor is inactive and by bisection where it binds; both paths are covered by the shipped automated tests (test_worked_example.py).

*Source:* code/calculate_scop_en14825.py, batch results in scop_validation/, and code/worked_example_two_stage.py in the data deposit.

## S-D. Stage-A bin decomposition table (worked example, 55 °C, Mode B)

Full EN 14825 Colder-bin decomposition of the worked example (cold-only coefficients, linear application curve, $\alpha_{\mathrm{bu}}$ = 1.048696). Reference bin loads $Q_{j}=p_{l,j}\cdot11.0$ kW; capacity split between the declared points $P_{\mathrm{HP}}(T_{\mathrm{OL}})$ = 8.0 kW and $P_{\mathrm{HP}}(T_{\mathrm{biv}})$ = 8.97 kW. Column sums: $E_{\mathrm{HP}}$ = 8,177.4 kWh, $E_{\mathrm{bu}}$ = 139.3 kWh; bin heat 27,112.4 kWh; full-system bin integral 27,112.4/8,316.7 = **3.2600** = declared.

| $T_{j}$ (°C) | $h_{j}$ (h) | $p_{l,j}$ | $T_{\sup}$ (°C) | $\Delta T$ (K) | $\mathrm{COP}_{\mathrm{pop}}$ | $\mathrm{COP}_{\mathrm{anch}}$ | $Q_{HP,j}$ (kW) | $Q_{bu,j}$ (kW) | $E_{HP,j}$ (kWh) | $E_{bu,j}$ (kWh) |
| --- | --- | --- | --- | --- | --- | --- | --- | --- | --- | --- |
| -22 | 1 | 1.0000 | 55.00 | 77.00 | 1.2626 | 1.3241 | 8.000 | 3.000 | 6.04 | 3.00 |
| -21 | 6 | 0.9737 | 54.17 | 75.17 | 1.3340 | 1.3989 | 8.139 | 2.572 | 34.91 | 15.43 |
| -20 | 13 | 0.9474 | 53.33 | 73.33 | 1.4072 | 1.4757 | 8.277 | 2.144 | 72.92 | 27.87 |
| -19 | 17 | 0.9211 | 52.50 | 71.50 | 1.4824 | 1.5546 | 8.416 | 1.716 | 92.03 | 29.17 |
| -18 | 19 | 0.8947 | 51.67 | 69.67 | 1.5597 | 1.6356 | 8.554 | 1.288 | 99.37 | 24.47 |
| -17 | 26 | 0.8684 | 50.83 | 67.83 | 1.6391 | 1.7189 | 8.693 | 0.860 | 131.48 | 22.35 |
| -16 | 39 | 0.8421 | 50.00 | 66.00 | 1.7209 | 1.8047 | 8.831 | 0.432 | 190.85 | 16.84 |
| -15 | 41 | 0.8158 | 49.17 | 64.17 | 1.8052 | 1.8931 | 8.970 | 0.004 | 194.27 | 0.15 |
| -14 | 35 | 0.7895 | 48.33 | 62.33 | 1.8921 | 1.9842 | 8.684 | 0.000 | 153.18 | 0.00 |
| -13 | 52 | 0.7632 | 47.50 | 60.50 | 1.9817 | 2.0782 | 8.395 | 0.000 | 210.05 | 0.00 |
| -12 | 37 | 0.7368 | 46.67 | 58.67 | 2.0744 | 2.1754 | 8.105 | 0.000 | 137.86 | 0.00 |
| -11 | 41 | 0.7105 | 45.83 | 56.83 | 2.1701 | 2.2758 | 7.816 | 0.000 | 140.81 | 0.00 |
| -10 | 43 | 0.6842 | 45.00 | 55.00 | 2.2693 | 2.3798 | 7.526 | 0.000 | 135.99 | 0.00 |
| -9 | 54 | 0.6579 | 44.17 | 53.17 | 2.3720 | 2.4875 | 7.237 | 0.000 | 157.10 | 0.00 |
| -8 | 90 | 0.6316 | 43.33 | 51.33 | 2.4787 | 2.5994 | 6.947 | 0.000 | 240.54 | 0.00 |
| -7 | 125 | 0.6053 | 42.50 | 49.50 | 2.5895 | 2.7156 | 6.658 | 0.000 | 306.47 | 0.00 |
| -6 | 169 | 0.5789 | 41.67 | 47.67 | 2.7048 | 2.8365 | 6.368 | 0.000 | 379.43 | 0.00 |
| -5 | 195 | 0.5526 | 40.83 | 45.83 | 2.8250 | 2.9626 | 6.079 | 0.000 | 400.12 | 0.00 |
| -4 | 278 | 0.5263 | 40.00 | 44.00 | 2.9505 | 3.0942 | 5.789 | 0.000 | 520.16 | 0.00 |
| -3 | 306 | 0.5000 | 39.17 | 42.17 | 3.0818 | 3.2319 | 5.500 | 0.000 | 520.75 | 0.00 |
| -2 | 454 | 0.4737 | 38.33 | 40.33 | 3.2195 | 3.3762 | 5.211 | 0.000 | 700.65 | 0.00 |
| -1 | 385 | 0.4474 | 37.50 | 38.50 | 3.3641 | 3.5279 | 4.921 | 0.000 | 537.04 | 0.00 |
| 0 | 490 | 0.4211 | 36.67 | 36.67 | 3.5163 | 3.6875 | 4.632 | 0.000 | 615.44 | 0.00 |
| 1 | 533 | 0.3947 | 35.83 | 34.83 | 3.6771 | 3.8561 | 4.342 | 0.000 | 600.17 | 0.00 |
| 2 | 380 | 0.3684 | 35.00 | 33.00 | 3.8473 | 4.0347 | 4.053 | 0.000 | 381.69 | 0.00 |
| 3 | 228 | 0.3421 | 34.17 | 31.17 | 4.0283 | 4.2244 | 3.763 | 0.000 | 203.10 | 0.00 |
| 4 | 261 | 0.3158 | 33.33 | 29.33 | 4.2212 | 4.4268 | 3.474 | 0.000 | 204.81 | 0.00 |
| 5 | 279 | 0.2895 | 32.50 | 27.50 | 4.4278 | 4.6434 | 3.184 | 0.000 | 191.32 | 0.00 |
| 6 | 229 | 0.2632 | 31.67 | 25.67 | 4.6502 | 4.8766 | 2.895 | 0.000 | 135.93 | 0.00 |
| 7 | 269 | 0.2368 | 30.83 | 23.83 | 4.8907 | 5.1289 | 2.605 | 0.000 | 136.64 | 0.00 |
| 8 | 233 | 0.2105 | 30.00 | 22.00 | 5.1526 | 5.4035 | 2.316 | 0.000 | 99.86 | 0.00 |
| 9 | 230 | 0.1842 | 29.17 | 20.17 | 5.4399 | 5.7048 | 2.026 | 0.000 | 81.69 | 0.00 |
| 10 | 243 | 0.1579 | 28.33 | 18.33 | 5.7578 | 6.0382 | 1.737 | 0.000 | 69.90 | 0.00 |
| 11 | 191 | 0.1316 | 27.50 | 16.50 | 6.1133 | 6.4109 | 1.447 | 0.000 | 43.12 | 0.00 |
| 12 | 146 | 0.1053 | 26.67 | 14.67 | 6.5160 | 6.8333 | 1.158 | 0.000 | 24.74 | 0.00 |
| 13 | 150 | 0.0789 | 25.83 | 12.83 | 6.9798 | 7.3197 | 0.868 | 0.000 | 17.80 | 0.00 |
| 14 | 97 | 0.0526 | 25.00 | 11.00 | 7.5256 | 7.8920 | 0.579 | 0.000 | 7.12 | 0.00 |
| 15 | 61 | 0.0263 | 24.17 | 9.17 | 8.1864 | 8.5850 | 0.289 | 0.000 | 2.06 | 0.00 |
| **Σ** | 6446 |  |  |  |  |  |  |  | **8177.40** | **139.28** |

*Source:* code/worked_example_two_stage.py in the data deposit.

## S-E. Heating-curve and rating-schedule sensitivity

**Rating-schedule identity.** The outlet-water temperatures at which the Colder-zone part-load points are declared in the certificate corpus lie, to 0.01 K data rounding, exactly on the line $(T_{\mathrm{design}},T_{supply,design})\to(20,20)$: 55 °C class 55.00/49.17/42.50/35.00/30.83/26.67 °C at −22/−15/−7/+2/+7/+12 °C; 35 °C class 35.00/32.50/29.64/26.43/24.64/22.86 °C. The method’s default curve therefore coincides with the rating outlet-water conditions. The only free convention is the extrapolation below the −15 °C test point: anchoring with the −15 °C outlet held constant (instead of continuing the line to the operating-limit point, which the certificates also declare on the same line) changes α by −0.55% (55 °C) / −0.18% (35 °C). These bound the “rating-schedule vs application-curve” transfer.

**Table S-E1. Curve-shape and pin sensitivity of the worked example (Mode B, re-anchored on the rating bins with the changed curve; % change in Stage-B annual electricity vs the frozen baseline 9,938 kWh).** The “fixed-α” column keeps the baseline anchoring while changing only the application curve — the exposure a user incurs by *not* re-running Step 3 (computed under the single-anchor variant; the split between conventions changes these values by < 0.1 pp).

| Variant | Re-anchored | Fixed α |
| --- | --- | --- |
| Radiator curve n = 1.1 | +0.04% | +2.4% |
| Radiator curve n = 1.2 | +0.09% | +4.5% |
| Radiator curve n = 1.3 | +0.14% | +6.5% |
| Radiator curve n = 1.4 | +0.19% | +8.3% |
| Radiator curve n = 1.5 | +0.23% | +9.9% |
| Linear, pin (18, 18) | −0.31% | −4.2% |
| Linear, pin (22, 22) | +0.24% | +3.9% |

*Source:* validation/rating_outlet_schedule_check.py and code/worked_example_two_stage.py in the data deposit.

## S-F. Capacity formulation (Step 4)

The capacity interpolation $Q_{\mathrm{HP}}=min(Q,P_{HP,lin}(T))$ replaces an earlier fixed-share ramp $\Phi(T)=\Phi_{\mathrm{OL}}+(1-\Phi_{\mathrm{OL}})(T-T_{\mathrm{OL}})/(T_{\mathrm{biv}}-T_{\mathrm{OL}})$ with $\Phi_{\mathrm{OL}}=P_{\mathrm{HP}}(T_{\mathrm{OL}})/P_{design,h}$. The share form is exact only when $T_{\mathrm{OL}}=T_{\mathrm{design}}$ (at other operating limits the reference load at $T_{\mathrm{OL}}$ is $p_{l}(T_{\mathrm{OL}}) P_{design,h}$, not $P_{design,h}$) and does not respond to the actual load magnitude. In the worked example the two formulations differ by 1.3% in annual electricity (9,995 vs 9,863 kWh at identical anchoring) and by a factor ≈ 2.6 in backup heat (546 vs 212 kWh); the capacity form is the more faithful representation of the declared data and grows the backup share automatically under undersizing within the TOL–T_biv interpolation window; general undersizing above T_biv requires the application-specific extension described separately. An application-specific extension for deliberately undersized systems (bivalent temperature recomputed from the capacity/load intersection) is demonstrated in the related research article’s supplementary material.

*Source:* code/worked_example_two_stage.py in the data deposit.

## S-G. Between-product spread of the population shape, before and after anchoring

Per-product COP curves (fixed + random effects, 979 cold-model products) evaluated at four lifts, raw and after normalising each product to a common anchored seasonal level over the Colder 55 °C bins (each curve divided by its own bin-integrated seasonal factor, re-scaled to the population value 3.128):

**Table S-G1. Population COP percentiles (p10/p50/p90) and relative p10–p90 spread.**

| ΔT | Raw p10/p50/p90 | Raw spread | Anchored p10/p50/p90 | Anchored spread |
| --- | --- | --- | --- | --- |
| 25 K | 4.29 / 4.73 / 5.17 | 18.6% | 4.49 / 4.76 / 4.98 | 10.3% |
| 40 K | 3.03 / 3.23 / 3.45 | 13.2% | 3.20 / 3.25 / 3.30 | 3.2% |
| 55 K | 2.15 / 2.27 / 2.39 | 10.5% | 2.21 / 2.26 / 2.35 | 6.3% |
| 70 K | 1.40 / 1.54 / 1.69 | 19.1% | 1.40 / 1.53 / 1.72 | 21.1% |

Anchoring removes 44%/75%/40% of the between-product spread at 25/40/55 K and essentially none at 70 K: the normalisation pivots the curves near the load-weighted mean lift (≈ 40 K) while product shapes diverge at the cold extreme — which coincides with the edge of the fitted lift range. The per-product bin-integrated seasonal factor itself spans p10/p50/p90 = 2.94/3.11/3.31. For reference, the per-product COP band at the −22 °C/55 °C design corner spans approximately 1.04–1.52 (5th–95th percentile) before anchoring.

*Source:* validation/anchored_spread.py in the data deposit.

## S-H. External benchmark and system-integration check (compact)

**hplib benchmark.** Curve level, against 9,314 air/water units of the independently developed hplib library, derived from public Heat Pump Keymark certification data: the population curve lies inside the hplib interquartile range at the mid-lift standard points (e.g. A−7/W35 and A+2/W55); at the extreme corners the two data structures diverge (hplib’s per-product parameterisations reach their own COP floor at A−15/W55); mean signed difference to the hplib population median −0.02 COP. Building level, same load and climate: population-mean default within +2.5% of the hplib median unit’s annual electricity; the population Mean→P10 and P5→P95 bands both bracket it.

**IDA ICE consistency check.** Same Estonian single-family house, weather and delivered heat, reference plant-control setting: space heating +0.1%, DHW −1.8%, total −0.9% (certificate-derived method vs dynamic on/off plant model). Temperature-resolved: +4.5% (conservative) below 0 °C — the range that carries 46% of annual electricity — and −5.4% above. Across seven plant-control settings the total spans −11…+12%, driven mainly by the DHW electric top-up control. Single-case model-consistency check; not a general validation.

*Source:* validation/ scripts and the IDA ICE energy-result files in ida_ice_check/ in the data deposit.

## S-I. Floor-activation thresholds and the Step-3 numerical solve

Declared-SCOP threshold below which the COP floor becomes active within the anchoring bins (the closed-form α then no longer reproduces the target and the monotone numerical solve takes over):

| Template / supply | Cold-only | All-data |
| --- | --- | --- |
| Colder, 35 °C | 1.92 | 1.97 |
| Colder, 55 °C | 2.48 | 2.49 |
| Average, 35 °C | 1.59 | 1.64 |
| Average, 55 °C | 1.91 | 1.93 |

Under the Mode-B (backup-aware) anchor of the worked example the Colder/55 °C threshold is 2.47. In all eight combinations the bisection solve converges and reproduces the seasonal target to |error| ≤ 10⁻¹³, and recovers the closed-form α wherever the floor is inactive (verified to 3 × 10⁻¹⁵ at the example’s declared 3.26). At a declared SCOP of 2.0 (floor active for Colder/55 °C), the solve still reproduces the target exactly.

*Source:* code/worked_example_two_stage.py and code/test_worked_example.py in the data deposit.

## S-J. Certification-condition backup share in the declared population

Motivation for the two-mode anchoring (Step 3): the backup-electricity share embedded in the declared Colder-zone SCOP, computed from the batch recalculation’s bin-wise supplementary-energy terms for the 5,503 valid air-to-water configurations:

| Statistic | Share of certification electricity |
| --- | --- |
| Median | 3.0% |
| Mean | 5.9% |
| 90th percentile | 16.5% |
| 95th percentile | 17.1% |
| Configurations above 1% / above 2% | 90.6% / 78.9% |

The share is driven by the declared operating limit: units with $T_{\mathrm{OL}}\leq-22{}^{\circ}C$ (n = 2,980) have median 2.8% and p90 3.4%, while units with $-22<T_{\mathrm{OL}}\leq-15{}^{\circ}C$ (n = 2,515) have median 10.9% and p90 17.5% (their sub-$T_{\mathrm{OL}}$ bins are met entirely by backup in the rating calculation). The worked-example unit’s own shares are 1.7% (55 °C) and 0.7% (35 °C). Because the share is material for a large part of the population, Mode B subtracts it from the anchoring target rather than counting it twice; Mode A retains it in the effective level, where nothing is added twice.

*Source:* validation/backup_share_population.py in the data deposit.

## S-K. External laboratory hold-out: WPZ Buchs published test results

**Source and cohort.** The WPZ heat-pump test centre (OST – Ostschweizer Fachhochschule, Buchs; EN ISO/IEC 17025-accredited) publishes a cumulative list of laboratory test results for air-to-water heat pumps (edition 29 May 2026, 19 pages). The list was transcribed with an automated table parser and checked against the PDF; every COP is a measured value (heating capacity / electrical input), not a manufacturer declaration. Sections A/B (54 units, tests 2015–2025) report EN 14511 full-load points and the EN 14825 part-load points of the Average climate at 35 °C and 55 °C application; sections C/D (54 units, tests 2006–2014, EN 14511:2004/2011) report full-load points only. After collapsing five rebrand duplicates (same test number under two holders), 104 tested units from 49 manufacturers remained, with 1,042 COP points, all inside the fitted lift range (ΔT 12–65 K).

**Screening against the training corpus.** WPZ is a registered Keymark test laboratory, so its reports can underlie certificates. Every unit was therefore screened against the frozen 18 March 2026 training corpus with the same identity cascade as the temporal hold-out (normalised manufacturer incl. OEM aliases, normalised model name, certificate identifiers, measurement signature). Two units returned an identity signal (one name-containment hit, one near-signature) and were excluded from the primary cohort; 77 units come from manufacturers absent from the training corpus (tier N-A) and the 51 units tested before 2015 predate the Keymark scheme. Primary cohort: 102 units, 49 manufacturers, 1,021 points. Analysis rules (cohort, strata, metrics) were written and hash-sealed before any residual was computed; no point or unit was selected by its agreement with the model; the frozen model was not refitted.

**Table S-K1. External laboratory hold-out errors (frozen cold-only fixed effects; point-weighted; signed error = predicted − observed; 95% intervals: 500-replicate manufacturer-cluster bootstrap).**

| Set | Units | n | MAE [95% CI] | RMSE | Mean bias [95% CI] | Median bias |
| --- | --- | --- | --- | --- | --- | --- |
| Primary cohort (in-range 10.9–77 K) | 102 | 1,021 | 0.448 [0.391, 0.521] | 0.607 | −0.054 [−0.208, +0.080] | −0.017 |
| EN 14511 full-load points | 102 | 656 | 0.350 [0.313, 0.389] | 0.437 | +0.036 [−0.086, +0.149] | +0.059 |
| EN 14825 part-load points | 51 | 365 | 0.623 [0.524, 0.759] | 0.829 | −0.218 [−0.422, −0.016] | −0.143 |
| ΔT < 30 K | 102 | 360 | 0.688 | 0.871 | −0.222 | −0.230 |
| ΔT 30–55 K | 102 | 520 | 0.338 | 0.416 | +0.080 | +0.083 |
| ΔT > 55 K | 94 | 141 | 0.236 | 0.304 | −0.122 | −0.133 |
| Tier N-A (manufacturers absent from training; 39 mfrs) | 77 | 777 | 0.464 | 0.635 | −0.087 | −0.038 |
| Tier N-B (manufacturer in training, unit unidentified; 10 mfrs) | 25 | 244 | 0.396 | 0.509 | +0.050 | +0.070 |
| Sensitivity: all 104 units incl. the two identity hits | 104 | 1,042 | 0.451 | 0.612 | −0.064 | −0.019 |
| Identity-hit units only | 2 | 21 | 0.622 | 0.832 | −0.555 | −0.365 |

All-data fixed effects on the primary cohort: MAE 0.446, RMSE 0.604, mean bias +0.049. Model-level distribution (102 units): MAE median 0.39, IQR 0.28–0.53. Ratio to the cross-validation MAE (0.369): 1.21 overall, 0.95 for the full-load points.

**Reading.** The external laboratory points are centred on the frozen curve overall, and the full-load points agree within cross-validation error. The deviation is carried by the EN 14825 part-load points — reduced-capacity operation at low lift, where inverter units run at their best efficiency — of the newer units, in the same direction and of the same size as the level shift found in the temporal hold-out (S-B). As a secondary, exploratory split by test era, units tested before 2015 (mostly R407C/R404A, many fixed-speed) lie slightly below the curve (419 points, bias +0.141) and units tested 2015 or later above it (602 points, bias −0.190). This is an external laboratory check under standardised EN 14511/EN 14825 conditions — not field validation — and residual overlap with certification test reports cannot be excluded with certainty for the tier N-B units, which is why the screening result is reported in full.

*Source:* validation/wpz_holdout.py (replay), scop_validation/wpz_points.csv, wpz_units.csv, wpz_exclusion_cascade.csv, wpz_cohorts.csv, the sealed rule files in scop_validation/wpz_rules/, scop_validation/SOURCES_wpz.md and code/test_wpz_holdout.py in the data deposit. The WPZ source document is not redistributed.
